# Editorial: Interaction of cell subtypes in tumor microenvironment, and implications for immunotherapy

**DOI:** 10.3389/fimmu.2025.1600338

**Published:** 2025-04-29

**Authors:** Hong Jiang, Lingfeng Fu, Takatsugu Ishimoto, Tao Zhang, Jun Zhang

**Affiliations:** ^1^ Hepatobiliary and Pancreatic Surgery, Liaoning Province Cancer Hospital & Institute (Cancer Hospital of China Medical University), Shenyang, Liaoning, China; ^2^ Division of Carcinogenesis, The Cancer Institute, Japanese Foundation for Cancer Research, Tokyo, Japan; ^3^ Gastric Cancer Department, Liaoning Province Cancer Hospital & Institute (Cancer Hospital of China Medical University), Shenyang, Liaoning, China

**Keywords:** tumor microenvironment, immunotherapies, macrophage, T cell, cell subtype classification

The tumor microenvironment (TME) is a highly dynamic and heterogeneous network where immune, stromal, and malignant cells interact to modulate cancer progression and immunotherapy outcomes. The Research Topic *“Interaction of Cell Subtypes in Tumor Microenvironment and Implications for Immunotherapy”* presents a collection of 11 articles that systematically investigates the molecular and cellular interactions within the TME, with a focus on immune cell heterogeneity, metabolic reprogramming, mechanical signaling, and therapeutic interventions. The findings collectively highlight the complexity of TME regulation and provide critical insights for optimizing immunotherapy strategies.

One of the central themes in this Research Topic is the heterogeneity of immune cells within TME. Tumor-associated macrophages (TAMs), a predominant component of the TME, exhibit remarkable plasticity, transitioning between pro-inflammatory (M1-like) and immunosuppressive (M2-like) phenotypes in response to environmental cues. Our previous research has challenged this conventional “binary” classification and identified a subset of macrophages with mixed M1/M2 polarization in diffuse-type gastric cancer (DGC) (1). Similarly, in colorectal cancer, Qi et al. utilized single-cell RNA sequencing (scRNA-seq) to confirm the presence of the Spp1+ macrophage subtype (2). Wang et al. also employed scRNA-seq to identify two novel TAM subtypes, FCN1+ TAMs, which are associated with inflammatory responses, and CCL18+ TAMs, which promote metastasis. Functionally, FCN1+ TAMs secrete IL-1β and TNF-α, thereby enhancing CD8+ T cell activation, whereas CCL18+ TAMs exhibit high expression of PD-L1 and VEGF-A, facilitating immune evasion and tumor angiogenesis. Notably, TAM polarization is dynamically regulated by tumor-derived cytokines (e.g., M-CSF, IL-4) and metabolic intermediates (e.g., lactate, succinate). For example, M-CSF induces M2 polarization via STAT3 activation, while hypoxic conditions enhance lactate production, which stabilizes HIF-1α and reinforces M2 phenotypes (Wang et al.). However, the extent to which FCN1+ and CCL18+ TAMs subtypes overlap with the conventional M1 and M2 classifications warrants further investigation. Similarly, Regulatory T cells (Tregs), known for their immunosuppressive role, contribute to tumor immune evasion by inhibiting effector T cell activation. Tregs further contribute to immune suppression through non-coding RNAs (miR-155, FOXP3-AS1) that modulate their metabolic and transcriptional programs. FOXP3-AS1 competes with FOXP3 mRNA for translation initiation, upregulating IL-10 expression, while miR-155 targets SOCS1 to enhance Treg suppressive activity (Ma et al.).

Beyond cellular heterogeneity, this Research Topic emphasizes the impact of TME remodeling on immunotherapy resistance. Several articles demonstrate that extracellular matrix (ECM) stiffness plays a crucial role in regulating immune cell behavior. Increased ECM stiffness promotes M2-like TAM polarization and impairs dendritic cell maturation, leading to ineffective T cell activation (Guenther). Additionally, cancer-associated fibroblasts (CAFs)s, which are responsible for remodeling ECM, contribute to immune exclusion by creating physical barriers that prevent immune cell infiltration and by secreting immunosuppressive cytokines that further dampen immune responses. Mechanistically, CAFs enhance ECM stiffness by cross-linking collagen and elastin fibers through the TGF-β/LOX signaling pathway (Fergatova and Affara). Mechanically stiffened ECM activates integrin-β1/FAK signaling in CAFs, promoting tumor stemness (WNT5A activation) and metastatic potential. Moreover, CAFs and TAMs form a metabolic symbiosis: CAFs convert glutamine to α-ketoglutarate, fueling TAMs’ tricarboxylic acid cycle, while TAMs export lactate via MCT4 transporters to sustain CAF energy production (Wu et al.). This metabolic coupling enhances immunosuppression and induces drug resistance through epigenetic modifications (e.g., histone acetylation via LDHA) (3). For instance, LDHA-catalyzed lactate dehydrogenase promotes histone acetylation of genes involved in drug resistance (e.g., ABC transporters). Additionally, senescent pancreatic cancer cells secrete CCL20, recruiting M2 macrophages and exacerbating immune suppression (Wu et al.).

Additionally, metabolic reprogramming within the TME, including lactate accumulation and hypoxia, further suppresses immune responses by inducing T cell exhaustion and promoting regulatory immune cell populations. A particularly novel finding is the role of tumor cell senescence in shaping the TME. In pancreatic cancer, senescent tumor cells secrete CCL20, which recruits M2 macrophages and enhances immune suppression (Wu et al.). Hypoxia and lactic acid accumulation could drive T cell exhaustion and M2 TAM polarization via the mTOR-HIF-1α axis. HIF-1α upregulates PD-1 expression on T cells and inhibits their mitochondrial function, while lactate activates NLRP3 inflammasomes to promote Treg expansion. ECM stiffness, mediated by PI3K/Akt signaling, further induces M2 TAM polarization and metastasis-associated gene expression (Guenther). In breast cancer models, hard collagen matrices increased MMP-9 expression and liver metastasis, whereas soft matrices suppressed these effects (4). These insights suggest that combining metabolic (e.g., LDHA/MCT4 inhibitors) and mechanical (e.g., PI3K inhibitors) interventions could synergistically reverse immune suppression.

To counteract these challenges, several emerging immunotherapeutic strategies have been proposed. One promising approach is targeting the Hippo signaling pathway, which influences macrophage polarization and immune cell differentiation. Hippo pathway inhibition enhances M1 TAM polarization by downregulating YAP/TAZ transcription factors, thereby augmenting antitumor immunity (Liu et al.). For example, YAP/TAZ knockout macrophages secreted elevated levels of IL-12 and IFN-γ, leading to enhanced CD8+ T cell infiltration and tumor regression (5). Another strategy involves integrating spatial transcriptomics with multi-omics profiling to define patient-specific TME signatures, which could guide the development of personalized immunotherapies (6). In melanoma patients, spatial metabolic analysis revealed that lactate accumulation correlated with reduced CD8+ T cell infiltration, suggesting MCT4-targeted interventions could improve treatment responses. Clinical trials demonstrated that triple checkpoint blockade (CD47-SIRPα+PD-1) achieved 70% complete remission in melanoma patients, while anti-IL-8 nanobodies increased CTLA-4 efficacy from 18% to 54% by blocking neutrophil infiltration (Tong et al.). These approaches hold promise for improving immune checkpoint blockade (ICB) responses and overcoming resistance mechanisms.

Despite these valuable contributions, several limitations remain. Current studies predominantly rely on xenograft models (>70%), which lack human-specific features such as neuro-immune interactions (Guenther). B cell dynamics, CD8+ T cell clonality, and immune temporal evolution remain underexplored, while static analyses limit understanding of TME dynamics. Another challenge is the variation in experimental methodologies across studies, making it difficult to standardize findings and draw definitive conclusions. Experimental variability across systems (cell lines/PDX/organoids) and technologies (scRNA-seq/spatial metabolomics) hinders cross-validation. Future efforts should integrate 4D spatiotemporal omics (live imaging + single-cell temporal analysis) with AI-driven predictive models (e.g., TME-DynaPredict) to monitor TME evolution in real time. Patient stratification strategies, such as proteinase inhibitor therapy for high-senescence-risk pancreatic cancer patients (Wu et al.), aim to reverse chemoresistance. Targeted delivery systems (e.g., nanobodies) could enable precise TME reprogramming, advancing immunotherapy toward precision medicine (7, 8).
